# Detection of Preventable Fetal Distress During Labor From Scanned Cardiotocogram Tracings Using Deep Learning

**DOI:** 10.3389/fped.2021.736834

**Published:** 2021-12-03

**Authors:** Martin G. Frasch, Shadrian B. Strong, David Nilosek, Joshua Leaverton, Barry S. Schifrin

**Affiliations:** Heart Rate AI, Inc., Seattle, WA, United States

**Keywords:** cardiotocography, deep learning-artificial neural network (DL-ANN), fetal brain injury, convolutional neural network (CNN), prevention

## Abstract

Despite broad application during labor and delivery, there remains considerable debate about the value of electronic fetal monitoring (EFM). EFM includes the surveillance of fetal heart rate (FHR) patterns in conjunction with the mother's uterine contractions, providing a wealth of data about fetal behavior and the threat of diminished oxygenation and cerebral perfusion. Adverse outcomes universally associate a fetal injury with the failure to timely respond to FHR pattern information. Historically, the EFM data, stored digitally, are available only as rasterized pdf images for contemporary or historical discussion and examination. In reality, however, they are rarely reviewed systematically or purposefully. Using a unique archive of EFM collected over 50 years of practice in conjunction with adverse outcomes, we present a deep learning framework for training and detection of incipient or past fetal injury. We report 94% accuracy in identifying early, preventable fetal injury intrapartum. This framework is suited for automating an early warning and decision support system for maintaining fetal well-being during the stresses of labor. Ultimately, such a system could enable obstetrical care providers to timely respond during labor and prevent both urgent intervention and adverse outcomes. When adverse outcomes cannot be avoided, they can provide guidance to the early neuroprotective treatment of the newborn.

## Introduction

In the United States, there are approximately four million births per year (1). Over 85% of them are accompanied by electronic fetal monitoring (EFM) in labor with the objective of safeguarding fetal/neonatal well-being. This surveillance of the FHR pattern (rhythm) in conjunction with the mother's uterine contractions provides a wealth of data about fetal behavior and the threat of diminished oxygenation and cerebral perfusion. Fifty years after its introduction, however, fetal monitoring continues to inspire debate about its value and especially its role in the increasing cesarean section rate as well as being a “litogen"—a stimulus to allegations of medical malpractice (2–10). Reviews of adverse labor outcomes in numerous countries universally associate adverse fetal outcomes with the failure to timely respond to the FHR pattern information [(11, 12); Inquiries, personal communication]. Indeed, various sources affirm that misinterpretation of EFM (or the uncertainty with patterns) has contributed to the significantly increased use of cesarean delivery from 5% in the 1970s to >30% today (13, 14), leading to increased expenditures, incurring costs in the United States (13, 14) of over $1 billion per year per 5% of additional cesarean deliveries (15). Obstetrical liability costs the country ~$40 billion per year, of which 70% is accounted for by uncertainty about EFM interpretation and related brain injury (14, 15).

Earlier and more precise recognition of the precursors of fetal compromise and the institution of corrective/preventative initiatives during labor are urgently needed. Only rarely should urgent delivery be required (16). Additional benefits include better maternal and child outcomes thanks to the avoidance of early intervention, lower cesarean delivery rate, and immediate neonatal monitoring of heart rate pattern, i.e., having the baby continuously monitored for at least 15 min after delivery. Here, babies seen to be at risk can be evaluated and more aggressively treated earlier than currently undertaken.

**Figure 1:**
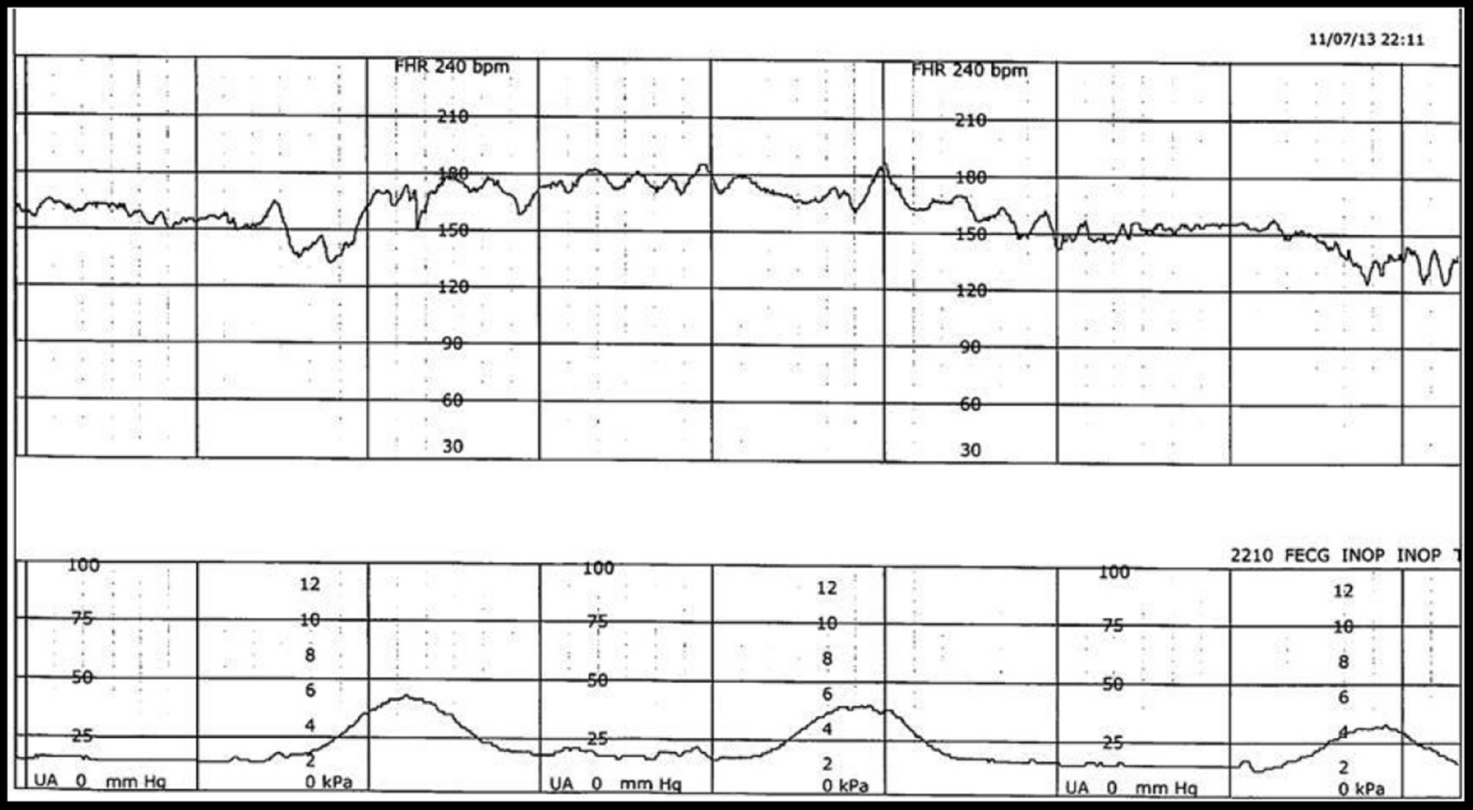
Example of FHR (top) and uterine contraction (bottom) during labor, captured simultaneously and stored electronically in a digital format but available only as a rasterized pdf document.

Historically, the EFM data, stored digitally, are available only as rasterized pdf images for contemporary or historical discussion and examination (Figure 1). In reality, however, they are rarely reviewed systematically or purposefully. In the case of a medical–legal review, it is the paper copy of the tracing, exclusively, that is likely available and consulted.

We propose a deep learning (DL)-based approach to this challenge. It is based on a unique archive which collected over four decades of EFM tracings of babies with known, adverse outcomes. This archive provides many unique examples of the broad range of healthy, threatened, and injured fetuses along with their long-term follow-up. Consequently, this archive is ideal for automating an early warning (preventive guidance) system for maintaining fetal well-being during the stresses of labor and delivery that could ultimately enable a health care provider to timely and conservatively respond during labor to prevent urgent interventions and adverse outcomes. When adverse outcomes cannot be avoided, they guide the early neuroprotective treatment of the newborn. This system utilizes a unique classification of heart rate and contraction patterns (details in section Methods), including specific identifiable indicators (“point A” and “point B”) of the need for attention by the provider (16–19).

## Methods

### Data

**Figure 2:**
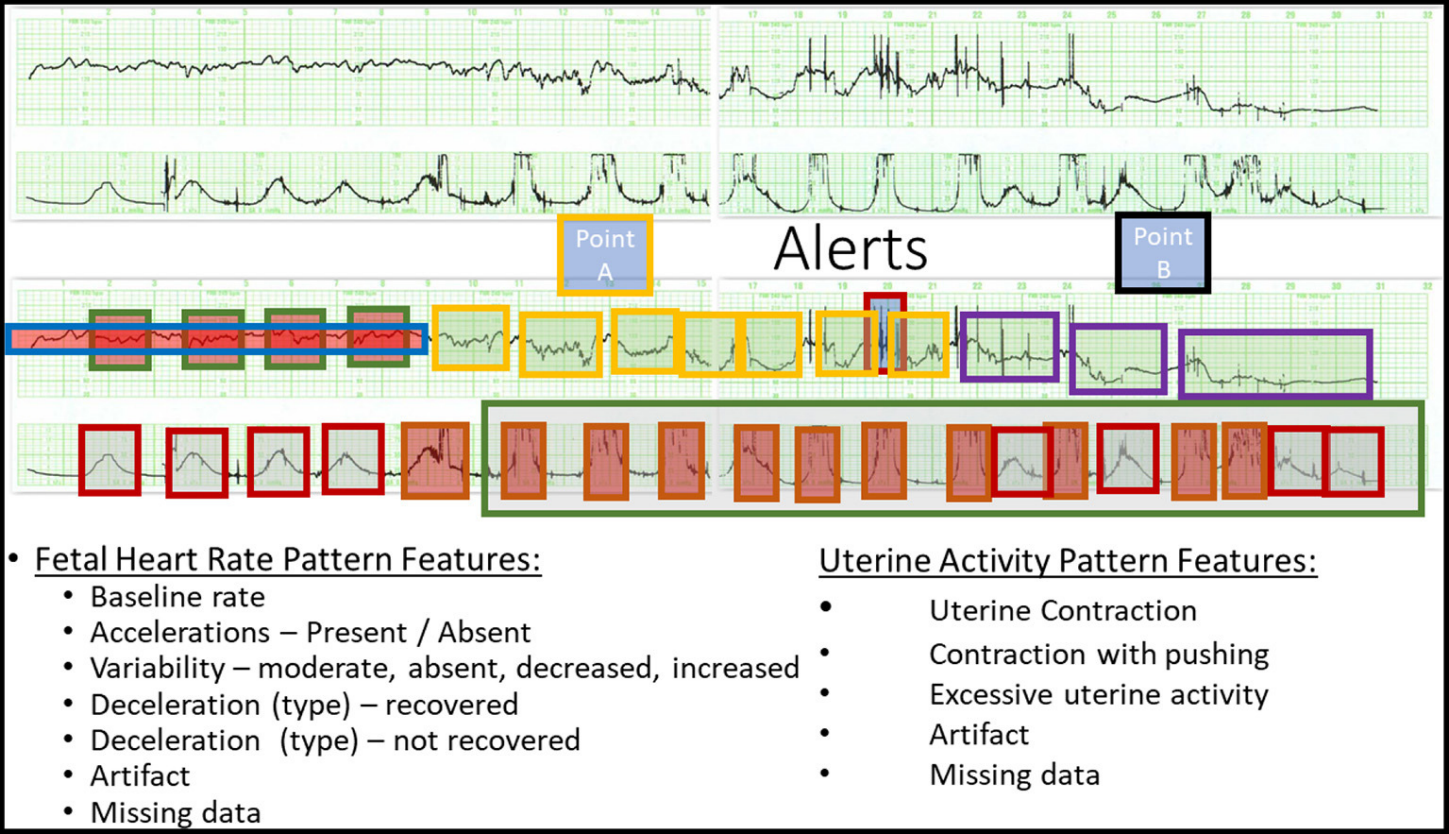
Definition of Point A and Point B. (Top) A representative raw CTG tracing. (Bottom) The annotated CTG tracing deriving Point A and Point B. See Tables 1, 2 for details.

For this pilot study, a convenience sample of 36 tracings was selected. All tracings were derived from singleton pregnancies at term undergoing a trial of labor with a fetal monitor in place as previously described (18). Each tracing was considered normal at the onset of monitoring—an important distinction. The majority of features were derived from conventional guidelines (ACOG) including baseline rate, variability, accelerations, and decelerations. For this study, however, certain operational definitions of heart rate patterns (Table 1) and uterine contractions (Table 2) were modified by the subject matter expert (Figure 2). These included the basal rate, the use of relative bradycardia and tachycardia, and the pattern of recovery of the deceleration.

**Table 1:** Definitions of EFM patterns.

Basal heart rate	The baseline FHR established at the beginning of labor with fetus quiescent
Tachycardia	Absolute—sustained (>10 min) baseline heart rate above 155 bpm
	Relative—sustained (>10 min) baseline heart rate >15 above basal rate
Bradycardia	Absolute—sustained (>10 min) baseline heart rate below 110 bpm
	Relative—sustained (>10 min) baseline heart rate >15 bpm below the basal rate
Deceleration recovery	The response of the fetus to a deceleration
	Categories of recovery:
Normal response	Prompt return to the previously normal baseline rate and variability
Adverse response	Applies to the recovery of the deceleration but may persist as a feature of the subsequent baseline heart rate
Overshoot	An acceleration of the FHR immediately following a deceleration with a duration proportional to the amplitude of the preceding deceleration. Usually associated with alterations in baseline rate and variability
Delayed return	A “slow return” to the baseline—likely a sustained elevation of fetal blood pressure in anticipation of recovery
Peaked return	An abrupt peak at the end of a deceleration followed by a late deceleration. An ominous commentary usually leading to fetal death
Decreased/absent variability	Persistent diminution in baseline variability <6 bpm
Increased variability	Persistent or transient elevation of variability >25 bpm
Sinusoidal pattern	Visually apparent, smooth, sine wave-like undulating pattern in FHR baseline with a cycle frequency of 3–5 per min. Occurs in the absence of a normal CTG pattern nearby. May be brief or persistent
Checkmark pattern	A unique pattern seen in neurologically compromised/asphyxiated fetuses suggesting repetitive “checkmarks” () of varying duration—frequently elicited by a preceding deceleration
Sawtooth pattern	Rapid, high frequency (20+ cpm), low amplitude (<15 bpm), peaked oscillations in the heart rate that generally increase in frequency and decrease in amplitude over time
Conversion	A CTG pattern in which there is a dramatic change in rate, variability, and pattern of deceleration within 1–2 contractions—suggests fetal ischemic injury

**Table 2:** Definition of excessive uterine activity.

**Contraction parameter**	**Average**	**Excessive**
Frequency	2–4.5 UC/10 min	>5/10 min (×2)
Intensity	25–75 mmHg	Not defined
Duration	60–90 s	>90 s
Resting tone	12–20 mmHg	>20 mmHg
Interval between peaks	2–4 min	<120 s
Rest time^*^	50–75%	<50%
Montevideo units	Not used	

* *Rest time—interval when contractions and pushing are absent*.

*UC, uterine contractions; mmHg, millimeters of mercury*.

### Identification of EFM Features

Tracing is defined at the outset of monitoring as normal or abnormal.

A normal tracing is characterized by a stable baseline heart rate between 110 and 155 bpm, with moderate variability and absent decelerations. An abnormal tracing is characterized by at least one of the following features:

- baseline heart rate: <100, >155, arrhythmia, unstable/indeterminate;
- baseline variability: absent, decreased (<6 bpm), increased (>25 bpm); and
- decelerations: late, variable, undefined.

Thus, for decelerations with normal recovery, no immediate action is required. They return promptly to the previously normal baseline variability (5–15 bpm peak to trough and chaotic, pseudorandom) and heart rate (usually 110–155 bpm and stable); each fetus has an individually unique baseline (basal rate). A “normal” deceleration returns to baseline without changing trajectory, and upon reaching the previous baseline rate remains there. These features pertain regardless of amplitude, duration, and timing of the deceleration and signify the comfortable compensation for the alteration in blood flow represented by the deceleration.

#### Point A

Point A denotes the time when the recovery of the deceleration is no longer “normal” and those additional compensatory activities are invoked by the fetus to maintain homeostasis. The detection of Point A signifies that increased attention and conservative measures are needed in an attempt to restore homeostasis to the previously normal tracing. These features include the following:

A. Delayed return to baseline: includes a change in the trajectory of the recovery such that the return to baseline is delayed beyond the end of the contraction.
B. Period of increased variability: peak to trough >20 bpm, frequency 5–10 cycles per min. Duration is also influenced by the appearance of a subsequent contraction during which time the pattern disappears—taken over by the deceleration.
C. Overshoot: an acceleration following the upslope of the return of the deceleration lasting 15 s or more prior to the return to the baseline.
D. Transient (usually at least 1 min) return to a higher baseline by at least 15 bpm, duration affected by next contraction, compared to the previously stable baseline.
E. Transient (at least 1 min) return to a lower baseline by at least 10 bpm compared to the previously normal baseline.
F. Excessive uterine activity—irrespective of changes in FHR pattern.

The detection of Point A alerts the health care provider to the need for at least conservative intervention in regard to the maternal condition, the frequency of contractions, or expulsive efforts during the second stage of labor. Point A is identified sooner if an excessive uterine activity is present.

#### Point B

Point B represents the attempt to define significant fetal compromise or injury, irrespective of the perceived amount of acidosis (pH) in the fetus. No clinical circumstances are used in the definition of Point B. Point B was identified by the subject expert (BSS) *via* a custom-created digital interface (AWS) allowing us to feed the annotations directly into the DL model.

These features include the following:

- sustained return to a baseline with diminished/absent baseline variability, usually accompanied by a rise in the baseline heart rate; and
- sustained change in baseline rate and variability with adverse features (Table 1) occurring within 5 to 10 min of a previously normal rate and variability—usually with recurrent variable decelerations.

### DL Pipeline

We present a method for automated extraction of features in FHR and uterine contractions (UCs), which are outlined in the above section.

Briefly, the method includes acquiring a set of non-digitized charts, digitally assigning markers to predetermined features in the charts, supplying the assigned marker-feature sets to a supervised model, statistically iterating over the assigned sets, automatically assessing model performance, and applying the model to new sets of charts to extract non-assigned predetermined features.

The method for automated chart processing includes analyzing time-series sets of non-digitized charts of FHR and/or concurrent maternal UC to digitally associate markers with fetal signatures, using the associated groups for supervised training of an artificial intelligence model, determining accuracy and precision of the model, and applying the trained model to automatically process new time-series sets of one or more charts of FHR and concurrent maternal UC, having one or more unassociated fetal signatures.

To achieve this goal, we treat scanned EFM recordings as non-vectorized images, similar to digital photographs, and apply supervised machine learning to extract and process features to train an artificial intelligence model. An image is supplied to a convolution neural network (CNN) model (20). The image is represented as one or more numeric arrays of pixel values with varying signal counts associated with the pixel content. The pixel content is dictated by the amount of red, green, blue, or other spectral bands that the pixel may receive and is an integer number in one or more dimensions. The CNN is represented as a set of algorithmic layers into which the numeric pixel data are sent. It consists of a series of convolutional layers, non-linear layers, pooling layers, and fully connected layers. Each such layer may be considered an individual set of equations, where the output of one equation becomes the input to another. The CNN eliminates the need for manual feature extraction, as the features are acquired through the passing of the pixel data to one or more other layers, and correlations are extracted and weighted as a consequence of the layer transitions.

We implement a single-shot detector (SSD) algorithm to achieve this goal (21). It utilizes a standard CNN network (e.g., VGG-16) with an additional set of convolution layers to identify discrete locations of one or more features in one or more images (22). The SSD codebase is available here: https://github.com/zhreshold/mxnet-ssd.

Through a single pass in the CNN, the weighted correlations meant to describe the relevant features are tested against ground truth data (validation data), separate from training data. The goal of this statistically iterative operation is to minimize a loss function between the predicted correlations and the truth values through adaptively updating the weights of the predicted function. The process of adjusting the weights continues until a minimum statistical loss is obtained.

The output model and weights are then used for inference against the withheld (unseen) dataset to extract similar relevant information.

#### Sample Selection and Processing

Briefly, in this study, we implemented a conventional random 80/20 train/test split. This corresponded to 26.4 h of training on EMF image information and 6.6 h used for testing (validation).

That is, the EFM images were flipped/translated, and the noise was added to represent more of the variability observed in the original pdfs.

The samples used in the analysis were 36 unique medical case reports in the form of a static pdf. The pdfs were split and converted into individual PNG images, one PNG per page in the pdf. As each pdf report consisted of a different number of unique pages, the number of images per page varied. In the end, there were 252 image pages across the 36 individual medical cases. The images were further cropped automatically to contain just the graphical data component of the page, removing headers, footers, and extraneous text. This was then split into 80/20 train/test datasets, resulting in 202 training image graphs and 50 test image graphs. Of the 50 test images, 25 were held back for separate validation. The images consolidated in these training and testing datasets were similar in quality (bold graphs with discernible FHR features). It should be noted that many additional pdf reports contained a varying degree of quality based on the photocopied/scanned/faxed nature of the captured data. This presented a significant challenge to create a robust training dataset with representative features. With 202/50 train/test data, significant augmentation was required. The images were flipped in horizontal space, as that preserved the domain of the information. A vertical flip would manifest in features unrepresentative of the FHR signatures. A further augmentation was required to reduce or sharpen the resolution of the images, to better capture the variability of the pdf graphs. In the end, 808 images were used in training and 200 in testing. These data are still rather shallow for DL, as the feature space and EFM signatures possible are vast. In future work, further data must be included in order to fully represent the feature space of the variabilities of both the documents and the EFM signatures. As an SSD algorithm was leveraged to isolate the EFM events, the images were manually annotated with standard data labeling practices, and output into the Pascal VOC XML format (http://host.robots.ox.ac.uk/pascal/VOC/).

## Results

The demographics and clinical characteristics are summarized in Table 3. There were 11 outcomes with a pH <7.00. Table 3 also denotes the duration and timing of the first and second stages of pushing, Point A and Point B.

Table 3Cohort characteristics.

**Table:** 

	**Age, years**	**EGA, weeks**	**BMI**	**BWT, g**	**Apgar 1**	**Apgar 5**
Median	26.5	39.8	31.0	3,325	2	6
25th	21.0	39.1	26.7	3,070.0	1.0	4.0
75th	30.3	40.4	35.9	3,601.8	4.0	7.0

**Table:** 

	**Temporal characteristics of labor (h:min)**						
	**1st**	**2nd**	**Labor**	**Point A**	**Point A to Delivery**	**Point B**	**Point B to Delivery**
Median	14:24	3:35	23:10	13:38	4:01	10:30	0:43
25th	1:30	2:49	16:33	5:44	1:52	4:20	0:23
75th	13:30	5:20	10:12	20:43	5:18	18:20	1:37

There were numerous points in the dataset that were abnormal but did not trigger Point A. Isolated but persistent changes in baseline rate, baseline variability, and excessive uterine activity are commonplace and do make the tracing abnormal without evoking Point A. Eventually, Point A was reached in all instances in this dataset. As such, from a machine learning perspective, this is a balanced dataset. This is also implied by the column “Point A to delivery” (Table 3).

**Figure 3:**
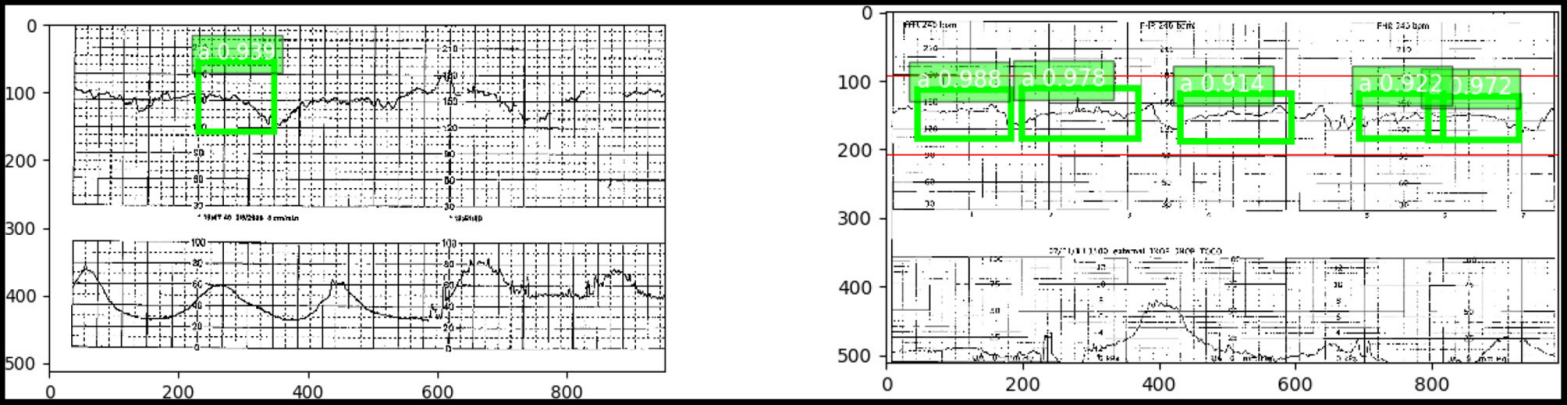
(Left) “Point A” identified with a 93.6% accuracy using an SSD trained on the pdf chart images. (Right) Numerous occurrences of “Point A” with high confidence in green. Red indicates the true “Point A” duration.

As a step toward developing this proactive fetal surveillance system, we have created an artificial intelligence model using a basic SSD DL approach to retrospectively identify critical features in the EFM data (cardiotocography) from the rasterized pdf directly (Figure 3). This model creates a classification of the pattern and identifies critical features of the tracing that indicate critical and timely points of either conservative or operative intervention, “Point A” and “Point B." Here, in the initial implementation, we focused on predicting “Point A.”

This novel application of using pdf rasterized plots as an image detection DL problem facilitates (1) quick and efficient deployment against a large record of data without chart digitizing and (2) packaging and deployment as a lightweight or MobileNet (23) application useful for immediate integration with a mobile device, post event.

The model achieved an accuracy of 93.6% in identifying Point A (i.e., detecting accurately the entire test set of features comprising Point A) against a small dataset with limited variability in features.

The average intersection over union (IoU) for the 25 validation images was 0.67, indicating a 67% overlap in the area with the true annotated feature. Annotated features are described in Table 1. This was averaged over 47 EFM bounding box features (true features and negative features) in the 25 images. Of the 47 features, the precision and recall were 87 and 82.5%.

## Discussion

Our primary goal was the early identification of abnormal tracings at the outset (considered Point A) and the early detection of isolated adverse features (abnormal) whose coalescence (Point A) demands intervention at a time when correction is likely. We successfully implemented automated identification of Point A, indicating threatened fetal decompensation of the highest relevance for real-time clinical implementation of such an algorithm. The SSD approach we deployed uses baseline data to identify Point A. In other words, the expert diagnosis of Point A on which the model was trained takes the baseline into account and seeks to identify the patterns comprising Point A in relation to the baseline.

In response to Point A, conservative rehabilitative measures include the following:

- diminishing the frequency of uterine contractions;
- diminishing/ceasing pushing during the second stage of labor;
- decreasing infusion of oxytocin; and
- assessing the feasibility of safe vaginal delivery.

However, the suggested measures cannot be ranked in relation to the probability score of Point A that our model provides as their sequence is primarily responsive to the feature(s) that prompted the response. If the problem involves excessive uterine activity, the care provider is directed to diminish uterine activity. If the response reveals late decelerations, the care provider is directed to modify the patient's position, providing supplemental oxygen, assisting with maternal blood pressure, etc.

Based on our observations of ~5,000 cases with brain injury as the birth outcome, 20% of normal patients reach Point A. About 25% of these revert to normal. Point B is reached in about 0.5% of the population and in about 30–40% of our observations on brain-injured babies with subsequent handicaps. We leave it to future work to implement the prediction of Point B, as this will require training on larger datasets. These points, together with other key signs in the FHR, can be displayed for the obstetrical care provider as part of an early alert and decision support system. Consequently, the visual signature for training the SSD is extracted similarly to the method utilized by the physician. The time-series nature of the FHR may be exploited with an additional application of a long short-term memory (LSTM) (24) model for consistent identification and tracking as a function of event duration. However, to date, only the SSD has been deployed.

It is important to emphasize that the training of the model was not based on the detection of acidosis or even low Apgar score, but whether or not conservative intervention based on the cardiotocographic pattern (Point A) was deemed necessary and whether criteria were met for the presumptive diagnosis of fetal neurological injury (Point B) as described previously (17). There was no attempt to correlate the outcome results with either pH or Apgar score of the newborn.

It may be seen as a limitation of the study that we did not seek correlations with fetal acidosis, Apgar scores, need for resuscitation, or NICU admission for HIE. However, the objective of our study was to use DL to prevent urgent intervention (“rescue”) by identifying the point in the previously normal tracing before fetal acidosis has developed and where conservative measures can be expected to restore the tracing to normal. We see no benefit in employing an artificial intelligence system to detect acidosis and, simultaneously, the need to rescue the fetus that may have already become injured. The system is designed to work with fetuses with initially normal tracings as no real benefit can be calculated from an algorithm that begins with an abnormal tracing where the options for prevention are limited and early delivery is likely (25).

Another limitation is that here we deliver a proof of concept only, using a convenience sample of 36 tracings in singleton pregnancies only. We leave a validation on the larger dataset and in multiple pregnancies, preterm deliveries, or IUGR fetuses for future work.

The approach to presenting and interpreting existing clinical data and annotating the EFM record during labor can dramatically reduce the need for urgent deliveries and significantly improve the outcomes of babies and mothers in labor and for the neonate in the nursery. Improved outcomes, less urgency, fewer rescues, and better documentation could be a game-changer for the care of pregnant women and children and the defense of allegations of obstetrical malpractice.

In future work, to boost the present performance results, alternatively or additionally, RCNN, LSTM, RNN, support vector machine, random forest, instance segmentation, image classification techniques, and/or other DL algorithms and/or other machine learning techniques can be applied.

The new EFM data can be supplied to the trained model in a format different from the format of the original training/testing images. For example, the EFM data can be supplied in the format of digitized charts, tabularly represented data, a signal received from one or more devices, etc. In other words, once the model has been trained, it can be configured to work on similar features provided in the same and/or other data formats, including live data. Such an approach allows the model to identify one or more features of interest and also the location of those features in the chart(s). This location can be correlated with a time and/or other dependent variables within the chart and/or a set of charts.

These features of our approach make it attractive to electronic medical record and physiological monitoring applications well beyond EFM.

We have shown the feasibility of a DL approach to scan and detect the ability of the fetus to handle the trial of labor using standard FHR and uterine activity chart tracings presented to artificial intelligence in the form of images, the format in which the majority of such tracings are still stored and presented to the experts for the determination of the need for intervention and the timing of the fetal injury. Our DL approach detects these factors with over 90% accuracy (compared to expert scoring).

## Data Availability Statement

The SSD codebase is available here: https://github.com/zhreshold/mxnet-ssd.

## Author Contributions

MGF, SS, DN, and BS conceived the manuscript. MGF wrote the initial draft. BS, SS, and MGF conducted the analyses. MGF, SS, DN, JL, and BS revised and approved the final version of the manuscript. All authors contributed to the article and approved the submitted version.

## Conflict of Interest

MGF, SS, DN, JL, and BS are co-founders of Heart Rate AI Inc. The research has been conducted as part of product development by Heart Rate AI Inc. and all work has been made open-source.

## Publisher's Note

All claims expressed in this article are solely those of the authors and do not necessarily represent those of their affiliated organizations, or those of the publisher, the editors and the reviewers. Any product that may be evaluated in this article, or claim that may be made by its manufacturer, is not guaranteed or endorsed by the publisher.
